# Research Insights on ‘Problematic Use of Short Video’: A Comprehensive Review Exploration in Psychological Context

**DOI:** 10.1111/adb.70082

**Published:** 2025-11-05

**Authors:** Tongshu Li, Huafang Liu, Run Hu, Xiaolong Liu

**Affiliations:** ^1^ Institute of Brain and Psychological Sciences Sichuan Normal University Chengdu China

**Keywords:** behavioural addiction, brain imaging, mechanism, problematic use of short video (PUSV), TikTok

## Abstract

Problematic use of short video (PUSV) refers to a psychological dependency on short‐video applications, characterized by an intense attachment and inability to control usage, resulting in typical addiction symptoms. Although PUSV shares similarities with substance addiction, its clinical presentation exhibits significant differences. Notably, the Diagnostic and Statistical Manual of Mental Disorders, Fifth Edition (DSM‐5), and the International Classification of Diseases, 11th Revision (ICD‐11), have not yet officially classified PUSV as an addiction disorder. However, the profound impact of short‐video platforms on individuals' physical, mental and daily lives necessitates attention to PUSV. To enable more in‐depth and standardized research on this issue, synthesizing and organizing existing studies on its theoretical foundations and mechanisms is crucial. This paper aims to integrate and evaluate existing research on PUSV from four perspectives: qualitative analysis, underlying mechanisms, negative effects and measurement tools, providing a more systematic understanding and approach to this emerging issue.

## 1 Introduction

The exponential growth of the internet and the ubiquitous embrace of smartphones have engendered a profound transformation in user engagement patterns, particularly with the advent of short‐video applications. Characterized by attributes of brevity, accessibility and immediacy, these short videos have swiftly ascended to prominence as a mainstream mode of new internet content dissemination over the past decade, deftly filling users' fleeting moments of leisure. However, alongside this trend, there has been an emergence of users engaging in uncontrolled and excessive use of short‐video applications, leading to detrimental effects on their physical and mental health, daily lives and their learning and work. This phenomenon has become more prevalent since the introduction of the short‐video application TikTok in 2016, attracting widespread attention from psychologists both domestically and internationally.

**FIGURE 1:**
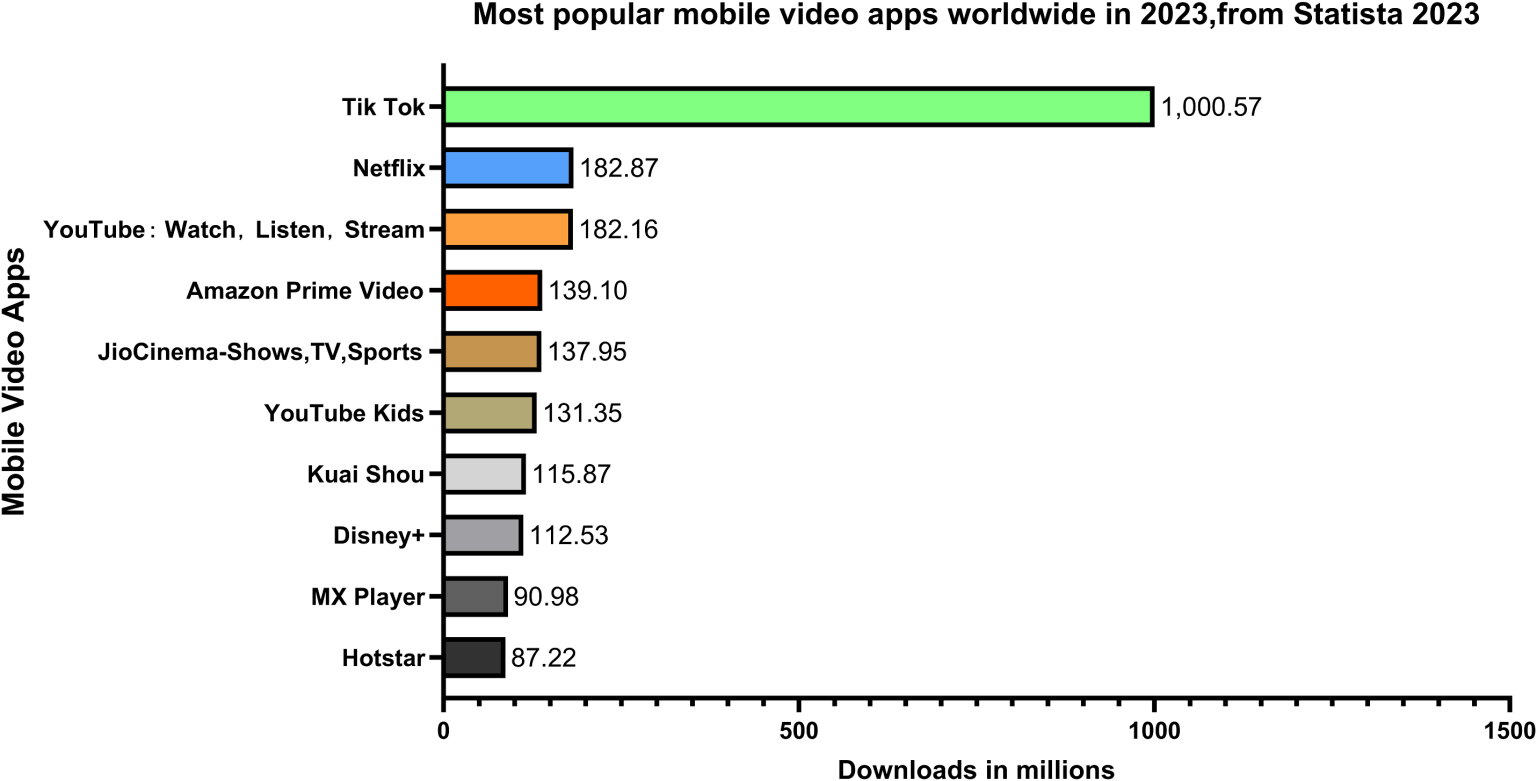
Global mobile video app download rankings, 2023 (Source Statista).

Since its launch in 2016, TikTok has played a key role in this phenomenon—expanding overseas in 2017 and consistently topping app store charts in over 40 countries. Within just 1 year, TikTok became the most popular global app with 150 million daily active users and 45.8 million downloads by June 2018 [1]. By 2019, TikTok had been downloaded nearly 1 billion times across 150 markets and 75 languages worldwide [2]. As per Statista 2023 data, TikTok emerged as the world's leading mobile video application in 2023 with over a billion downloads, significantly surpassing Netflix's second‐place figure of 182 million global downloads (Figure 1).

Behind the surge in popularity, short‐video applications have also given rise to certain issues. Some users find it challenging to exercise self‐control and end up excessively using these applications, leading to negative impacts on their mental health (such as anxiety and depression) [3], daily life (including social activities and sleep quality) [4] and academic or professional pursuits (like distraction, reduced study time and diminished time management ability) [5]. Due to the resemblance of these problems with clinical manifestations of substance addiction, many researchers have characterized this issue as ‘behavioural addiction’ and termed it ‘Short Video Addiction’ (SVA). Existing studies indicate that SVA shares similarities with other social media application addictions. For instance, some scholars have suggested that short‐video addiction conceptually resembles social media addiction based on research findings related to Facebook addiction [6]. Subsequently, building upon the concept of social media addiction, subsequent researchers proposed a specific definition for ‘short video addiction’, which involves a strong attachment and compulsive repeated use of short videos leading to typical symptoms of addiction in individuals [7].

As the concept of ‘short video addiction’ continues to be refined, many researchers have begun to explore the mechanisms of ‘short video addiction’. Researchers have proposed explanations for SVA based on various theories, including Uses and Gratifications Theory [8], Life History Strategies (LHS) [9] and Opponent Process Theory (OPT) [10]. However, there is no unification and continuous innovation in explaining the mechanism of SVA, and most of the theories used are borrowed from existing addiction theories or related research theories. In addition to theoretical mechanisms, researchers have also relied on neuroimaging devices to study the brain activity of short‐video addicts, finding that watching TikTok's personalized short videos continues to stimulate the ventral tegmental area (VTA) of the user's brain, which may be the neural basis of ‘short video addiction’ [11]. The VTA is a key area of neural circuitry involved in pleasure and motivational reinforcement, and high and sustained activation of this area provides pleasure and motivational reinforcement to individuals, reducing their self‐control and ultimately leading to persistent craving for short‐video use and ‘short video addiction’. The findings are also consistent with other existing brain region activation findings in behavioural addictions (e.g., internet addiction) [12, 13], online gaming addiction [14] and smartphone addiction [15].

Moreover, due to the numerous negative effects of ‘short video addiction’, the mechanisms of these problems have also been extensively investigated by researchers, such as the effects of SVA on users' attention and time management skills [5]; the effects of SVA on users' mental health problems (anxiety and depression) [3, 16]; and the effects on users' real‐life social interactions and interpersonal relationships [4]. The impact of overuse and problematic use of short video, and the extensive attention it has received from researchers, has made this research an increasingly important cutting‐edge topic in contemporary psychology. Nevertheless, considering that the issue of short‐video addiction is not adequately addressed in the International Classification of Diseases, 11th Revision (ICD‐11), Addictive Behaviour Branch, nor in the DSM‐5 utilized in the United States, we will use the term ‘Problematic Use of Short Video’ to describe this problem more rigorously. To further advance research in this area, this paper focuses on the literature related to short‐video addiction, problematic short‐video use and short‐video use disorder. By integrating and evaluating the existing research on ‘Problematic Use of Short Video’ from four perspectives: qualitative, mechanism, negative effects and measurement tools, we aim to provide a more systematic understanding and approach to this emerging issue.

## 2 The Concept and Qualitative Nature of ‘Problematic Use of Short Video’—Problematic Use or Behavioural Addiction?

The classification of short‐video use as an addiction has been debated. On one hand, ‘problematic use of short video’ has not yet been included in the International Classification of Diseases, 11th Revision (ICD‐11), Addictive Behaviour Branch, or in the DSM‐5 in the United States, which means that problematic use of short video has not yet been recognized as an ‘addiction problem’ in the official classification of mental disorders and conditions. On the other hand, the symptoms of problematic short‐video use differ from those of substance and behavioural addictions that have already been included in the classification. However, the symptoms of problematic short‐video use exhibit some similarities with those of substance addiction and behavioural addiction, leading many researchers to prefer the term ‘short video addiction’ (SVA) to describe this phenomenon [17, 18].

### 2.1 The Concept of ‘Problematic Use of Short Video’

The definition of ‘problematic use of short video’ varies among studies that support the addiction perspective. Tian et al. [19] defined it from the perspective of technology addiction, arguing that ‘short video addiction’ is an addiction to short‐video technology and should be classified as a branch of behavioural addiction. Conversely, Zheng posited that ‘short video addiction’ should be considered a subtype of internet addiction, significantly impacting users' reality adaptation and well‐being [20]. Mu et al. [21] provided a more comprehensive definition based on existing studies, describing it as a strong, uncontrollable urge to repeatedly use a short‐video application, leading to typical addiction symptoms such as withdrawal (significant negative emotions when unable to access the short‐video application), salience (the overwhelming impact of short videos on one's thoughts and tasks), conflict (interference with the user's other activities) and relapse (inability to reduce the time of use). It is important to note that the pathology of short‐video addiction or problematic use of short video is not officially recognized, and the concept lacks consensus and clear criteria among researchers [22].

### 2.2 The Qualitative Nature of ‘Problematic Use of Short Video’

According to the medical definition, ‘addiction’ refers to the replacement of physical dependence on a substance with long‐term withdrawal symptoms such as fatigue, anxiety and vomiting when the substance is discontinued [23]. The DSM‐5 classifies four criteria for the diagnosis of substance addiction: ‘control disorder’, ‘social disorder’, ‘substance use harm’ and ‘pharmacological harm’. At least two to three criteria must be met to determine the existence of the corresponding disorder. In contrast, the ICD‐11 states that the main clinical features of behavioural addiction include repetitive patterns, impaired control, addictive behaviours and the presence of a substance abuse disorder.

#### 2.2.1 Salience

Salience is the symptom most easily demonstrated in behavioural addictions, meaning that the behaviour becomes the most important activity in an individual's life and dominates their thoughts, feelings and behaviours [24]. Research on problematic use of short video indicates that short videos occupy a significant portion of users' fragmented daily time, demonstrating salience. Due to their personalized push and high entertainment value, many users quickly progress to problematic use of short video and become highly dependent on them. Statistics show that teenagers represent the highest proportion of short‐video users in China, with many reporting frequent usage: 20.49% of Chinese teenagers (aged 13–18) say they watch TikTok anytime, anywhere; 32.69% use it multiple times a day; and only 18.75% say they use it infrequently [25]. Another survey found that the majority of adolescents use TikTok for up to 3 h per day, with some using it for 3 to 10 h, and instances of usage exceeding 10 h have been reported. Additionally, many adolescents admitted to staying up late to use TikTok [26]. According to Marengo et al. [27], 40% of adolescents between the ages of 14 and 17 use social media like TikTok for more than 4 h per day. Semistructured interviews analysing addiction indicators among young short‐video users revealed that respondents reported that short‐video use can easily evolve into a daily habit, often used in conjunction with other activities (e.g., working out, taking a walk), with daily usage often exceeding 4 to 5 h [17]. All these data and studies support the notion that short videos meet the significance indicators regarding users' life time domination and occupation.

#### 2.2.2 Mood Modification

Mood modification reflects the fact that addictive behaviours can have different emotional moderating effects on the addict. For instance, smoking addicts may smoke in the morning for refreshment and in the evening for relaxation after a hard day's work [24]. Griffiths suggests that for addicts, the psychological effects of addictive behaviours are usually greater than the physiological effects; these behaviours become a reliable and persistent way for addicts to alter their emotional state, eventually developing into a self‐medicating coping strategy.

Research on problematic use of short video indicates that mood modification is particularly evident. From the perspective of Uses and Gratifications Theory, users engage with short videos because they satisfy diverse personal needs and interests [8]. Structured interviews revealed that users derive considerable satisfaction from short videos, leading to emotional regulation. TikTok users reported, ‘I repeatedly watch a certain type of short video because I find the video really interesting’; ‘I prefer watching short videos to regular videos because they are often within one minute or only ten seconds, which satisfies my need to watch’; and ‘I know there is a push mechanism for short videos that keeps pushing me the videos I like, but also so that every video is interesting and makes me want to keep watching’ [17]. These user feedback results demonstrate that short videos provide significant emotional value, such as interest and satisfaction of needs, proving that they can alleviate negative emotions and play a role in emotional regulation.

#### 2.2.3 Tolerance

Tolerance is demonstrated when addicts need to increase the amount of stimulation from an addictive substance or behaviour to achieve the effects previously experienced, a symptom commonly seen in drug addicts as a gradual increase in drug dosage [24]. Tolerance has also been observed in behavioural addictions. However, existing research on problematic use of short video has not specifically discussed the ‘tolerance’ aspect, and it has not been adequately demonstrated. Studies indicate that short‐video users tend to have individual differences in fixed daily use time, frequency of use and total use time, without showing significant trends of sudden increases in viewing time. For example, surveys of teenagers' TikTok usage show that most teenagers use the app for a stable time of about 3 h [26]. Additionally, short‐video users generally prefer using the app at night and during leisure time, with 91.3% of users preferring to use TikTok at night (19:00–7:00) [28].

#### 2.2.4 Withdrawal Symptoms

Withdrawal symptoms manifest as heightened arousal and discomfort when an addict is separated from the addictive substance or activity [29]. This can present itself psychologically as moodiness or physically as nausea, headache, sweating and insomnia [24]. Withdrawal symptoms have also been observed in behavioural addictions. Research on problematic use of short video indicates that withdrawal symptoms are one of its core symptoms [30]. Turel validated their model of technology addiction by extending the Perceived Enjoyment Model Test to technology addiction through a self‐constructed auction test. They identified ‘withdrawal’ symptoms as ‘negative emotions when the user is unable to use short videos’. Other studies have concluded that the withdrawal reaction of ‘short video addicts’ is pronounced, with users experiencing withdrawal symptoms when they are forced to stop using short videos or voluntarily cease usage, leading to difficulty in controlling their use to escape the withdrawal feeling [19].

#### 2.2.5 Conflict

Conflict refers to the discord between addicts and those around them (interpersonal conflict) or within the addict themselves (psychological conflict) [24]. Interpersonal conflict arises when the addicted person clashes with partners, relatives or friends, damaging their social relationships. Psychological conflict occurs when the addictive behaviour leads the individual to prioritize short‐term pleasure and relief, ignoring long‐term negative consequences. Existing research on problematic use of short video shows that conflict symptoms are evident. Short‐video applications expand users' online interpersonal interactions, but dependence on these applications negatively impacts offline social interactions [3]. Additionally, the time and energy consumed by problematic use of short video directly affect users' work efficiency [5], time management abilities and academic performance [31]. Furthermore, problematic short‐video use can lead to internal problems such as attention‐related issues, emotional and cognitive problems and mental health problems, including higher risks of depression and anxiety compared to nonaddicts [32].

#### 2.2.6 Relapse

Relapse refers to the rapid return of an addict's pre‐withdrawal behaviours after a period of abstinence, a symptom common in substance addictions [24]. Research on problematic use of short video indicates that relapse symptoms are evident. As an online media addiction, problematic short‐video use exhibits intermittent relapse [33]. Studies have found that addiction to online technologies, such as short videos, increases user satisfaction and promotes continued use, leading to a recurring cycle that deepens the addiction problem [34]. Users may rationalize their behaviour, exhibiting intermittent discontinuation, where they neither use the app as frequently as before nor completely give it up, but instead stop using it for a short period and then resume usage [35].

In summary, while problematic use of short video converges on most behavioural addiction symptoms, including salience, mood modification, withdrawal symptoms, conflict and relapse, there is a notable lack of research on tolerance, which is a significant symptom of substance addiction. This gap may be due to methodological limitations in studying this issue, such as reliance on cross‐sectional designs and self‐reported data, making it difficult to explore causality [8]. Combined with the above discussion of addiction symptoms, it can be concluded that compared with ‘short video addiction’, it may be more objective to refer to this issue as ‘problematic use of short video’.

## 3 Mechanisms for the Problematic Use of Short Video

According to existing research, the mechanisms of problematic short‐video use are mainly divided into theoretical mechanisms and neural mechanisms. Theoretical mechanisms primarily include three types: the ‘Uses and Gratifications Theory’ for the relationship between media and users, the ‘Life History Strategy Theory’ for the differences in individual life strategies of short‐video users and the ‘Opponent Process Theory’ based on the positive and negative reinforcement mechanism combined with the user's emotions. In terms of neural mechanisms, fMRI brain imaging has been used to observe the brain activity of short‐video addicts while watching short videos, proposing that the core area of addiction is the VTA of the brain.

### 3.1 Theoretical Mechanisms of Problematic Use of Short Video

#### 3.1.1 Uses and Gratifications Theory (UG)

Studies have developed models to explain the problematic use of short video based on the Uses and Gratifications Theory [36], which is the most widely used theory in media research. This theory suggests that users are positively reinforced by the gratifications and positive emotions that short‐video content providers, leading to an increase in the frequency and duration of use, ultimately making it difficult for users to disengage from short‐video applications [8]. The core of the UG theory revolves around ‘motivation and satisfaction’. Research has shown that core motivations for TikTok users include self‐expression, social interaction and escaping reality [1].

#### 3.1.2 LHS

Wang et al. combined the LHS with the S‐K selection strategy model to develop the ‘fast‐slow LHS strategy’ suitable for the problematic use of short video. Fast‐slow LHS posits that individuals who adopt a fast life strategy tend to focus on immediate interests and pleasure, making them more prone to problematic use of short video, which provide high short‐term excitement. Conversely, individuals with a slow life strategy are less likely to engage in problematic use due to their ability to delay gratification and consider long‐term consequences [9].

#### 3.1.3 OPT

OPT describes the emotions of short‐video users through an opposing mode. Existing studies explain the mechanism of problematic use of short video through the positive and negative emotional reinforcement of OPT. When users interact with short videos, pleasant events activate positive emotions, leading to positive reinforcement. However, when users are forced to stop using short videos, negative emotions arise, triggering a cycle where users seek to maintain positive emotions and minimize negative emotions through continued use [19].

### 3.2 Neural Mechanisms of Problematic Use of Short Video

Studies have shown that problematic short‐video users exhibit high activation in the VTA when watching personalized short videos. The VTA is a key neural circuit region involved in pleasure and motivation enhancement, and long‐term activation of this region can induce a desire for short videos and problematic use [11]. Research using functional magnetic resonance imaging (fMRI) has indicated that problematic short‐video users have lower activation in brain regions related to inhibitory control, suggesting that their ability to regulate usage is compromised when engaging with short videos. Additionally, the activation of the default mode network (DMN) during short‐video viewing indicates that personalized content engages a wide range of cognitive tasks and self‐referential processing, further reinforcing addictive behaviours.

## 4 Negative Effects of Problematic Use of Short Video

The negative effects of problematic use of short video can be categorized into behavioural effects and psychological effects.

### 4.1 Behavioural Effects of Problematic Use of Short Video

Research indicates that problematic use of short video negatively impacts users' attention and time perception. Problematic users exhibit lower levels of attentional focus, shorter gaze times, longer reaction times and higher error rates when processing information [3, 8]. Additionally, the immersive experience provided by short videos can interfere with individuals' time perception, leading to a distorted sense of time and reduced awareness of their surroundings [11].

### 4.2 Psychological Effects of Problematic Use of Short Video

Studies have shown that problematic use of short video is associated with increased mental health issues, including higher levels of depression, anxiety and stress compared to healthy users [32]. The immersive nature of short videos can lead to strong withdrawal reactions when users are unable to access the content, resulting in negative emotions such as anxiety and depression. Additionally, the phenomenon of Fear of Missing Out (FOMO) may contribute to mental health problems, as users experience anxiety about missing important information or social interactions when they are unable to engage with short videos [37].

## 5 Short‐Video Problematic Use Measurement Tools

The development of psychometric instruments for assessing short‐video addiction has lagged, with standardized scales and structured questionnaires gaining traction only recently. The primary tools for measuring short‐video addiction are often adaptations of existing addiction scales, such as those for smartphone and internet addiction, with less emphasis on neuroimaging devices.

### 5.1 College Students' Short‐Video Addiction Scale

In 2019, the ‘College Students' Short Video Addiction Scale’ was developed, based on the ‘Mobile Phone Addiction Index Scale’ (MPAI). This scale consists of four factors: withdrawal, escapism, loss of control and inefficiency, with a total of 14 items. It demonstrated good reliability and validity, indicating its potential for assessing short‐video addiction among college students.

### 5.2 TikTok Addiction Scale—Short Version

In 2022, the ‘TikTok Addiction Scale—Short Version’ was adapted from the ‘Smartphone Addiction Scale—Short Version’. This scale retains the original's six factors and 33 items, focusing on daily life interference, positive anticipation, escapism, online relational orientation, excessive use and tolerance. The revised scale showed positive reliability and validity results.

### 5.3 Short‐form Videos Problem Use Scale

In 2023, the ‘Short‐form Videos Problem Use Scale’ was created by adapting the ‘Mobile Phone Problem Use Scale’. The revised scale demonstrated good reliability and cross‐cultural applicability, validating its use for assessing short‐form video addiction.

## 6 Future Perspectives

Research into the problematic use of short video is still in its infancy. Conceptually, the problematic use of short video lacks a unified definition. While symptoms are similar to those of substance addiction, they do not fully meet the diagnostic criteria for behavioural addiction. Mechanistically, there is still room for exploration in both behavioural and neural mechanisms. Future studies should focus on defining the concept of short‐video problematic use more accurately and enriching research methodologies. This includes conducting longitudinal studies, incorporating qualitative methods and utilizing neuroimaging technology to analyse the neural mechanisms involved in attention, emotion and reward processes.

In conclusion, as a unique product of the new era, short videos with their algorithmic mechanisms can significantly satisfy user needs. However, the potential negative effects of short videos warrant further attention and research. The journey to understanding and addressing the problematic use of short video is ongoing.

## Author Contributions

**Tongshu Li:** conceptualization, methodology, validation and writing original draft. **Huafang Liu:** validation and formal analysis. **Run Hu:** validation and review. **Xiaolong Liu:** validation, review and editing, project administration and funding acquisition. All authors contributed to and approved the final version of the manuscript and take responsibility for the integrity of the data and the accuracy of the data analysis.

## Ethics Statement

The study and its procedures had full approval by the local ethics committee and adhered to the most recent version of the Declaration of Helsinki; all participants were required to provide informed consent.

## Conflicts of Interest

The authors declare no conflicts of interest.

## Data Availability

The data presented in this review are available upon request from the corresponding author.
